# Clinical Characteristics, Long‐Term Outcomes, and Prognostic Factors in High‐and Very High‐Risk Prostate Cancer: A 25‐Year Institutional Cohort Study

**DOI:** 10.1155/aiu/9913238

**Published:** 2026-08-17

**Authors:** Tomoyuki Shimabukuro, Hidekazu Tanaka, Masahiro Tanabe, Takanori Tokunaga, Kosuke Shimizu, Nakanori Fujii, Keita Kobayashi, Toshiya Hiroyoshi, Hiroshi Hirata, Koji Shiraishi

**Affiliations:** ^1^ Department of Urology, Graduate School of Medicine, Yamaguchi University, Ube, Yamaguchi, Japan, yamaguchi-u.ac.jp; ^2^ Department of Urology, Ube Central Hospital, Ube, Yamaguchi, Japan; ^3^ Department of Radiation Oncology, Graduate School of Medicine, Yamaguchi University, Ube, Yamaguchi, Japan, yamaguchi-u.ac.jp; ^4^ Department of Radiology, Graduate School of Medicine, Yamaguchi University, Ube, Yamaguchi, Japan, yamaguchi-u.ac.jp

**Keywords:** biochemical recurrence, ECOG performance status, high-risk prostate cancer, very high-risk prostate cancer

## Abstract

**Background and Objective:**

High‐ and very high‐risk prostate cancer (hvPCa) is associated with substantial prostate cancer–specific mortality, making optimal treatment selection challenging. This study reevaluated our 25‐year institutional experience with hvPCa to assess long‐term oncological outcomes and identify prognostic factors associated with survival.

**Methods:**

We retrospectively analyzed 220 patients with hvPCa reclassified according to the latest National Comprehensive Cancer Network guidelines. Of these, 66 underwent radical prostatectomy (RP), 52 received radiotherapy plus androgen deprivation therapy (RT + ADT), and 102 received primary ADT. Long‐term oncological outcomes were compared across treatment modalities and risk groups. Prognostic factors associated with prostate cancer–specific survival (PCSS) were evaluated using Cox proportional hazards models.

**Results:**

The median age was 71 years, and the median follow‐up duration was 88 months. During follow‐up, 49 patients (22.3%) died from any cause, and 19 (8.6%) died from prostate cancer. Among high‐risk patients, overall survival was significantly better in the RP group than in the ADT group (hazard ratio [HR]: 4.812; 95% confidence interval [CI]: 1.832–12.635; *p* = 0.001). Among very high‐risk patients, PCSS was significantly worse in the ADT group than in the RP group (HR: 2.899; 95% CI: 1.454–5.782; *p* = 0.003). Multivariable analysis identified ECOG performance status ≥ 2 (HR: 40.756; 95% CI: 1.846–899.767; *p* = 0.019) and biochemical recurrence (HR: 21.566; 95% CI: 1.079–430.937; *p* = 0.044) as independent predictors of poorer PCSS.

**Conclusions:**

Poorer ECOG performance status and biochemical recurrence were independently associated with worse PCSS in patients with hvPCa. These findings highlight the prognostic importance of patient functional status in addition to conventional tumor‐related factors and may contribute to improved risk stratification and individualized treatment selection.

## 1. Introduction

Recent clinical guidelines emphasize the importance of shared decision‐making in prostate‐specific antigen (PSA) screening. The National Comprehensive Cancer Network (NCCN) risk classification system for prostate cancer (PCa) incorporates key prognostic factors, including life expectancy, Gleason grade group, clinical stage, and PSA levels [1]. According to the current guidelines, definitive local therapy is recommended for patients with intermediate‐risk disease and a life expectancy of more than 10 years, as well as for those with high‐ or very high‐risk disease and an expected survival exceeding 5 years. However, despite advances in diagnosis and treatment, high‐ and very high‐risk PCa (hvPCa), regardless of regional lymph node involvement, remains associated with substantial PCa–specific mortality and accounts for nearly two‐thirds of PCa‐related deaths within 10 years [2].

A comprehensive evaluation of institutional experience with radical prostatectomy (RP), radiation therapy combined with androgen deprivation therapy (RT + ADT), and primary ADT for hvPCa may provide important insights into the long‐term effectiveness of these treatment strategies. In particular, assessment of oncological outcomes, including overall survival (OS) and PCa‐specific survival (PCSS), may facilitate evidence‐based treatment selection. Because treatment decisions for hvPCa are influenced not only by tumor characteristics but also by patient‐related factors such as age, comorbidities, and performance status, these variables should be carefully considered when comparing outcomes among treatment modalities.

The primary objective of this study was to reevaluate our 25‐year institutional experience in patients with hvPCa after reclassification according to the latest NCCN guidelines (version 2.2025). We also sought to investigate the prognostic impact of baseline clinical characteristics, including performance status, on long‐term oncological outcomes in this patient population.

## 2. Patients and Methods

### 2.1. Study Population

Between January 2000 and December 2019, 1278 patients with pathologically confirmed PCa were treated at Yamaguchi University Hospital and Ube Central Hospital. Follow‐up data were collected through December 2024. Among these patients, 220 were identified as having hvPCa and were reclassified according to the latest NCCN guidelines (Version 2.2025) [1]. The treatment cohort comprised 66 patients who underwent RP (52 high‐risk and 14 very high‐risk), 52 who received RT + ADT (30 high‐risk and 22 very high‐risk), and 102 who received primary ADT (39 high‐risk and 63 very high‐risk).

### 2.2. Data Collection and Quality Control

Baseline variables included age, serum PSA level, clinical T stage, Gleason grade group, NCCN risk category, and Eastern Cooperative Oncology Group (ECOG) performance status. ECOG performance status at treatment initiation was retrospectively extracted from medical records and categorized as 0‐1 or ≥ 2 for subsequent analyses. Clinical information was retrospectively collected from electronic and paper‐based medical records and supplemented by direct patient contact when necessary. Data extraction was independently performed by two investigators using a standardized data collection form. Any discrepancies were resolved by consensus. The final dataset was locked in March 2025. The median follow‐up duration was 88 months. All data were anonymized prior to analysis.

### 2.3. Statement of Ethics

The study protocol was approved by the Institutional Review Board of Yamaguchi University School of Medicine (No. H2020‐229) and Ube Central Hospital (No. 70160502). All procedures were conducted in accordance with the principles of the Declaration of Helsinki and relevant institutional guidelines. The requirement for written informed consent was waived because of the retrospective nature of the study.

### 2.4. Diagnostic and Treatment Procedures

Details regarding diagnostic evaluations, staging procedures, surgical indications, fiducial marker placement, intensity‐modulated radiation therapy (IMRT), and data collection have been reported previously [3]. In brief, all patients who underwent RP received extended pelvic lymph node dissection. Patients treated with RT + ADT underwent implantation of gold fiducial markers before IMRT (total dose: 78 Gy) and received 24 months of ADT. ADT primarily consisted of combined androgen blockade using a gonadotropin‐releasing hormone (GnRH) analog in combination with bicalutamide. During the latter part of the study period, selected patients additionally received androgen receptor pathway inhibitors.

### 2.5. Definitions of Biochemical Recurrence (BCR) and Castration‐Resistant PCa (CRPC)

BCR after RP was defined as a confirmed PSA ≥ 0.2 ng/mL [4]. For patients treated with RT + ADT, BCR was defined according to the Phoenix criteria as a PSA increase of ≥ 2 ng/mL above the nadir value, irrespective of concurrent hormonal therapy [5].

CRPC was defined according to PSA progression criteria. In patients who achieved a PSA decline, CRPC was defined as a ≥ 25% increase from the nadir and an absolute increase of ≥ 2 ng/mL confirmed at least 3 weeks later. For patients without an initial PSA decline after initiation of ADT, CRPC was defined according to PCWG3 recommendations as a ≥ 25% increase in PSA from baseline after at least 12 weeks of treatment, in the setting of castrate testosterone levels [6].

### 2.6. Statistical Analysis

Statistical analyses were performed using StatView Version 4.5 (SAS Institute, Cary, NC, USA). Categorical variables were compared using Fisher’s exact probability test, whereas continuous variables were analyzed using the Kruskal–Wallis rank test. OS and PCSS were estimated using the Kaplan–Meier method and compared using the log‐rank test. Variables considered clinically relevant or previously associated with survival outcomes were entered into the Cox proportional hazards models. These variables included age, ECOG performance status, baseline PSA level, clinical N stage, occurrence of BCR, ALP and LDH fluctuation patterns [7], and treatment modality.

All statistical tests were two‐sided, and *p* values < 0.05 were considered statistically significant.

## 3. Results

### 3.1. NCCN Risk Stratification for Clinically Localized Disease

Table 1 summarizes the modified NCCN risk stratification for high‐risk and hvPCa and the corresponding treatment recommendations according to estimated life expectancy [1].

**TABLE 1:** Modified National Comprehensive Cancer Network (NCCN) risk stratification for high‐ and very high‐risk prostate cancer.

Risk group	Clinical/Pathological features	Life expectancy	Treatment options
High	Has all of one or more high‐risk features, but does not meet criteria for very high risk:	> 5 years or symptomatic	RT plus ADT or RP with extended pelvic lymph node dissection
	• cT3 ‐ cT4	≤ 5 years and asymptomatic	RT, ADT, or observation
	• Grade Group 4 or Grade Group 5		
	• PSA > 20 ng/mL		

Very high	Has at least two of the following:	> 5 years or symptomatic	RT plus ADT along with or without ARPIs for 24 months or RP with extended pelvic lymph node dissection
	• cT3 ‐ cT4		
	• Grade Group 4 or Grade Group 5	≤ 5 years and asymptomatic	RT, ADT, or observation
	• PSA > 40 ng/mL		

Abbreviations: ADT, androgen deprivation therapy; ARPI, androgen receptor pathway inhibitor; PSA, prostate‐specific antigen; RP, radical prostatectomy; RT, radiation therapy.

### 3.2. Demographic and Clinical Characteristics

Baseline demographic and clinical characteristics are presented in Table 2. The median age of the entire cohort was 71 years (interquartile range [IQR]: 67–77 years). Age distribution did not differ significantly between the high‐risk and very high‐risk groups. However, significant differences in age distribution were observed among treatment groups within both risk categories. In the very high‐risk cohort, clinical N stage and baseline PSA level also differed significantly among treatment groups.

**TABLE 2:** Baseline demographic and clinical characteristics of study cohort.

		High‐risk cohort			*p* value	Very high‐risk cohort			*p* value
	All	RP	RT + ADT	ADT		RP	RT + ADT	ADT	
	(*n* = 220)	(*n* = 52)	(*n* = 30)	(*n* = 39)		(*n* = 14)	(*n* = 22)	(*n* = 63)	
Age, y					< 0.001				< 0.001
< 71	100	38	11	8		12	13	18	
≥ 71	120	14	19	31		2	9	45	
ECOG performance status					0.118				0.217
≤ 1	212	52	30	37		14	22	57	
≥ 2	7	0	0	2		0	0	5	
Clinical N stage					0.337				0.007
0	196	52	30	37		14	21	42	
1	19	0	0	1		0	1	17	
Baseline PSA (ng/mL)					0.269				0.023
median	36.5	26.4	27.6	28.9		44.8	45.8	72.6	
IQR	24.7–69.4	23.0–35.7	21.4–33.3	23.2–45.1		27.1–60.1	26.0–95.5	46.1–174.0	
PSA doubling time at BCR, months					NE				NE
≤ 9	18	12	1	NA		4	1	NA	
> 9	9	4	1	NA		4	0	NA	
Follow‐up characteristics									
Median follow‐up months	88.0	93.0	95.0	64.0	< 0.001	115.0	106.5	64.0	< 0.001
IQR	60.0–122.0	72.5–176.0	81.0–120.0	33.3–88.8		75.0–229.0	97.0–133.0	27.3–109.3	
Median months to BCR	33.500	38.000	83.000	NA	0.123	29.000	75.000	NA	0.091
IQR	18.0–70.5	17.5–70.3	76.0–90.0	NA		18.0–47.0	63.0–87.0	NA	
Median months to DM	33.5	NA	83.0	NA	NE	29.0	75.0	NA	NE
IQR	18.9–70.5		76.0–90.0			18.0–47.0	63.0–87.0		
Biochemical recurrence no. (%)					< 0.001				< 0.001
Present	36 (16.4)	21 (40.4)	2 (6.7)	NA		11 (78.6)	2 (9.1)	NA	
Distant metastases (DM) no. (%)					0.346				0.234
Present	4 (1.8)	0	1 (3.3)	0		2 (14.3)	1 (4.5)	0	
Mortality no. (%)									
All‐cause mortality	49 (22.3)	8 (15.4)	2 (6.7)	12 (30.8)	0.024	6 (42.9)	0	21 (33.3)	0.004
PCa‐specific mortality	19 (8.6)	2 (3.8)	0	2 (5.1)	0.469	3 (21.4)	0	12 (19.0)	0.071

*Note:* BCR, biochemical recurrence; PCa, prostate cancer; IQR, interquartile range; NE, nonestimable, representing no cancer‐specific deaths and/or monotone likelihood ratio; y, years.

Abbreviations: ADT, androgen deprivation therapy; DM, distant metastases; ECOG, Eastern Cooperative Oncology Group; NA, not applicable; PSA, prostate‐specific antigen; RP, radical prostatectomy; RT, radiation therapy.

Baseline serum testosterone levels (reference: 1.31–8.71 ng/mL) were comparable between younger (< 71 years) patients (median: 4.52 ng/mL; IQR: 2.92–5.70) and older (≥ 71 years) patients (median: 4.58 ng/mL; IQR: 3.54–5.90) (*p* = 0.660).

The median follow‐up duration for the entire cohort was 88 months (IQR: 60–122 months). Excluding patients treated with primary ADT, who frequently progressed directly to CRPC, BCR occurred in 19.0% of high‐risk patients (median time to BCR: 48 months) and 13.1% of very high‐risk patients (median time to BCR: 29 months), with significant differences among treatment groups (*p* < 0.001).

Four patients developed distant metastases, including one in the high‐risk group and three in the very high‐risk group. During follow‐up, 49 patients (22.3%) died from any cause, and 19 (8.6%) died from PCa.

### 3.3. OS and PCSS in High‐Risk Patients

**FIGURE 1:**
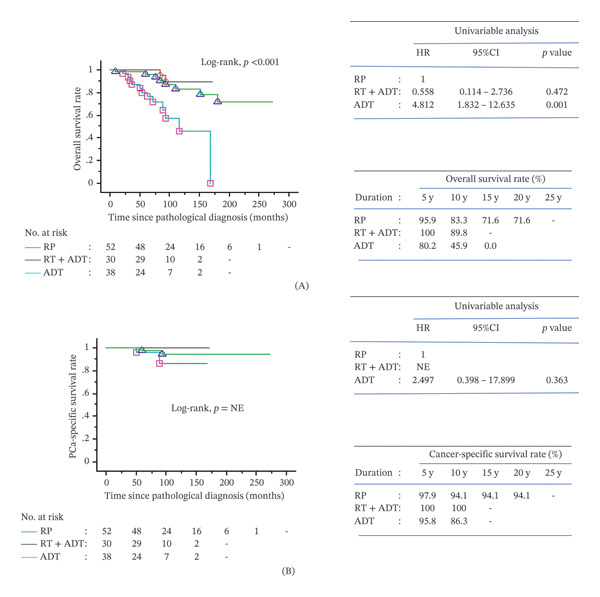
(A). Kaplan–Meier curves of overall survival in high‐risk prostate cancer patients. RP: radical prostatectomy; RT: radiation therapy; ADT: androgen deprivation therapy; HR: hazard ratio; CI: confidence interval; y: years. (B) Kaplan–Meier curves of prostate cancer–specific survival in high‐risk prostate cancer patients. RP: radical prostatectomy; RT: radiation therapy; ADT: androgen deprivation therapy; HR: hazard ratio; CI: confidence interval; NE: nonestimable; y: years.

Kaplan‐Meier curves for OS and PCSS in high‐risk patients are shown in Figures 1A and B, respectively. In univariable analysis, the ADT group demonstrated significantly poorer OS than the RP group (hazard ratio [HR]: 4.812; 95% confidence interval [CI]: 1.832–12.635; *p* = 0.001). No significant difference in PCSS was observed between these treatment groups. No PCa–specific deaths occurred in the RT + ADT group; therefore, PCSS analysis was not performed for this subgroup.

10‐year OS rates:

• RP: 83.3%
• RT + ADT: 89.8%
• ADT: 45.9% (median survival: 112 months)

10‐year PCSS rates:

• RP: 94.1%
• RT + ADT: 100%
• ADT: 86.3%

### 3.4. OS and PCSS in Very High‐Risk Patients

**FIGURE 2:**
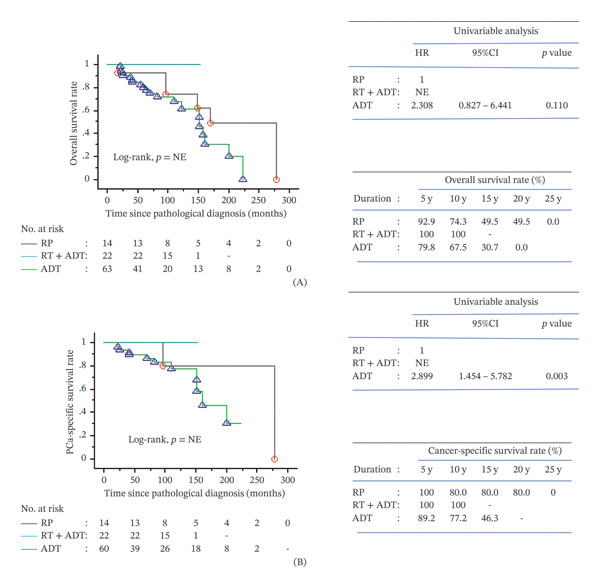
(A). Kaplan–Meier curves of overall survival in very high‐risk prostate cancer patients. RP: radical prostatectomy; RT: radiation therapy; ADT: androgen deprivation therapy; HR: hazard ratio; CI: confidence interval; NE: nonestimable; y: years. (B) Kaplan–Meier curves of prostate cancer–specific survival in very high‐risk prostate cancer patients. RP: radical prostatectomy; RT: radiation therapy; ADT: androgen deprivation therapy; HR: hazard ratio; CI: confidence interval; NE: nonestimable; y: years.

Kaplan‐Meier curves for OS and PCSS in patients with very high‐risk disease are presented in Figures 2A and B, respectively. In the univariable analysis, OS did not differ significantly between the RP and ADT groups (HR: 2.308; 95% CI: 0.827–6.441; *p* = 0.110). Similar to the high‐risk cohort, no PCa‐specific deaths occurred in the RT + ADT group. However, PCSS was significantly lower in the ADT group than in the RP group (HR: 2.899; 95% CI: 1.454–5.782; *p* = 0.003).

10‐year OS rates:

• RP: 74.3%
• RT + ADT: 100%
• ADT: 67.5%

10‐year PCSS rates:

• RP: 80.0%
• RT + ADT: 100%
• ADT: 77.2%

### 3.5. Risk Factors for PCa‐Specific Mortality in Very High‐Risk Patients

Table 3 summarizes the Cox proportional hazards analyses for PCSS in the very high‐risk cohort. In univariable analysis, older age, clinical N1 disease, and a lower alkaline phosphatase fluctuation after 1 year [7] were significantly associated with worse PCSS. Multivariable analysis identified ECOG performance status and BCR as independent predictors of PCa‐specific mortality. ECOG performance status ≥ 2 was associated with significantly poorer PCSS (HR: 40.756; 95% CI: 1.846‐899.767; *p* = 0.019). Likewise, the occurrence of BCR independently predicted worse PCSS (HR: 21.566; 95% CI: 1.079 – 430.937; *p* = 0.044).

**TABLE 3:** Cox proportional hazards and univariable and multivariable analysis of risk factors for prostate cancer–specific survival in very high‐risk patients.

	Univariable			Multivariable		
	HR	95% CI	*p* *value*	HR	95% CI	*p* *value*
Age, y						
< 71	1			1		
≥ 71	4.078	1.162–14.317	0.028	9.139	0.390–214.437	0.169
ECOG performance status						
≤ 1	1			1		
≥ 2	3.576	0.752–17.014	0.109	40.756	1.846–899.767	0.019
Baseline PSA (ng/mL)	1.001	0.998–1.004	0.377	1.002	0.997–1.006	0.467
Clinical N stage						
0	1			1		
1	3.624	1.081–12.152	0.037	5.573	0.666–46.644	0.113
BCR						
Negative	1			1		
Positive	0.497	0.107–2.303	0.371	21.566	1.079–430.937	0.044
ALP fluctuation pattern						
Inter QR	1			1		
Upper QR	1.401	0.331–5.933	0.647	3.655	0.296–45.159	0.312
Lower QR	5.686	1.031–31.352	0.046	8.198	0.322–208.781	0.203
LDH fluctuation pattern						
Inter QR	1			1		
Upper QR	2.452	0.612–9.824	0.205	3.918	0.279–55.111	0.311
Lower QR	1.250	0.228–6.863	0.797	0.502	0.030–8.292	0.630
Treatment methods						
RP	1			1		
RT + ADT	NE			NE		
ADT	2.899	1.454–5.782	0.003	NE		

*Note:* BCR, biochemical recurrence; ALP, alkaline phosphatase; LDH, lactate dehydrogenase; y, years.

Abbreviations: ADT, androgen deprivation therapy; CI, confidence interval; DM, distant metastases; ECOG, Eastern Cooperative Oncology Group; HR, hazard ratio; NE, nonestimable; PSA, prostate‐specific antigen; QR, quartile range; RP, radical prostatectomy; RT, radiation therapy.

Treatment methods were not estimable, representing no cancer‐specific deaths in the RT + ADT subgroup and monotone likelihood ratio in this cohort.

## 4. Discussion

Treatment options for patients with hvPCa include primary ADT, RP with extended pelvic lymph node dissection, and RT + ADT. However, optimal risk stratification and treatment selection remain subjects of ongoing debate. To date, no prospective randomized trials have directly compared these modalities in patients with hvPCa, and current clinical decision‐making therefore relies largely on retrospective evidence.

In the present study, we reevaluated a 25‐year institutional cohort according to the latest NCCN risk classification system and investigated long‐term oncological outcomes in patients with hvPCa. The overall mortality rate was 22.3%, whereas PCa‐specific mortality was 8.6% during a median follow‐up period of 88 months. Among patients with high‐risk disease, OS was significantly better in the RP group than in the primary ADT group, although no significant difference in PCSS was observed. In contrast, among patients with very high‐risk disease, OS did not significantly differ among treatment modalities, whereas PCSS was significantly worse in patients treated with primary ADT than in those treated with RP. Furthermore, multivariable analysis identified ECOG performance status and BCR as independent predictors of PCa‐specific mortality. These findings suggest that patient‐related factors may be as important as tumor‐related characteristics when evaluating prognosis in very high‐risk disease.

Although favorable outcomes were observed in both the RP and RT + ADT groups, interpretation of the RT + ADT results requires caution. No PCa‐specific deaths occurred among patients treated with RT + ADT; however, the sample size was limited, and the number of patients at risk decreased substantially during long‐term follow‐up. Moreover, all patients in the RT + ADT cohort received 24 months of ADT in combination with definitive radiotherapy. Because patients with high‐ and very high‐risk disease are at increased risk of occult nodal or distant metastatic disease, the systemic effect of ADT may have contributed to delaying disease progression and cancer‐specific mortality. Consistent with this possibility, the HR for OS in the RT + ADT group compared to the RP group was 0.558, although this difference did not reach statistical significance. Further follow‐up is warranted to clarify the long‐term comparative effectiveness of RT + ADT and RP in patients with hvPCa.

A notable finding of the present study was the independent prognostic significance of ECOG performance status in patients with very high‐risk disease. Traditionally, treatment selection and risk stratification in PCa have been based primarily on tumor‐related factors, including PSA level, Gleason grade group, and clinical stage. Our findings suggest that patient‐related characteristics also play an important role in determining long‐term outcomes. ECOG performance status may reflect not only physical functioning but also frailty, comorbidity burden, and the ability to tolerate intensive treatment. Therefore, assessment of performance status should be incorporated into treatment decision‐making and prognostic evaluation for patients with very high‐risk PCa.

Although the HR associated with ECOG performance status was substantial and the CI was wide because of the limited number of PCa‐specific deaths, the association remained statistically significant after multivariable adjustment. This observation supports the clinical relevance of performance status in this patient population. Our findings are consistent with previous reports in advanced PCa, in which ECOG performance status has repeatedly been identified as an independent predictor of survival outcomes [8, 9].

BCR was also identified as an independent predictor of PCa‐specific mortality. Management of patients who developed BCR following definitive local therapy remains controversial. Although PSA‐only recurrence is generally considered an imperfect surrogate for PCa‐specific or OS [4], a subset of patients with high‐risk BCR experience rapid disease progression and are at increased risk of cancer‐related deaths [10]. In our cohort, approximately two‐thirds of patients with BCR met the criteria for high‐risk BCR, defined by a PSA doubling time of 9 months or less. This finding highlights the biological aggressiveness of recurrent disease in patients with hvPCa.

Preventing or delaying clinical progression while avoiding overtreatment remains a major challenge for clinicians managing patients with BCR. Although adjuvant and salvage treatment strategies are available, the optimal approach remains uncertain and differs according to the primary treatment modality. Future advances in molecular diagnostics, genomic risk stratification, and next‐generation imaging techniques may improve the identification of patients at highest risk for progression and facilitate more individualized treatment strategies.

Several limitations of this study should be acknowledged. First, the study was retrospective and observational in nature and involved a relatively limited sample size. Second, treatment allocation was not randomized, resulting in unavoidable selection bias. Patients treated with ADT were generally older and may have had greater comorbidity burdens or poorer suitability for definitive local therapy. Third, physician preference and differences in posttreatment management may have influenced long‐term outcomes. Fourth, the introduction of IMRT in 2008 limited the duration of follow‐up available for patients treated with RT + ADT. Finally, treatment‐related complications, functional outcomes, and quality‐of‐life measures were not evaluated. Therefore, the findings of this study should be interpreted cautiously and require validation in larger prospective cohorts.

## 5. Conclusion

This long‐term retrospective study provides valuable insights into the clinical and oncological outcomes of patients with hvPCa. Among patients with hvPCa, ECOG performance status emerged as an independent predictor of PCSS. Together with BCR, patient functional status provided important prognostic information beyond conventional tumor‐related factors. These findings support the incorporation of performance status into individualized risk assessment and treatment selection for patients with high‐ and very high‐risk disease.

**Nomenclature**
ADT: Androgen deprivation therapy
BCR: Biochemical recurrence
hvPCa: High and very high–risk prostate cancer
OS: Overall survival
PCSS: Prostate cancer–specific survival
PSA: Prostate‐specific antigen
RP: Radical prostatectomy
RT: Radiation therapy

## Author Contributions

Conceptualization: Tomoyuki Shimabukuro. Project administration: Tomoyuki Shimabukuro and Koji Shiraishi. Data curation: Tomoyuki Shimabukuro, Takanori Tokunaga, Kosuke Shimizu, Nakanori Fujii, Keita Kobayashi, Toshiya Hiroyoshi, and Hiroshi Hirata. Formal analysis: Tomoyuki Shimabukuro. Writing–original draft: Tomoyuki Shimabukuro. Writing–review and editing: Tomoyuki Shimabukuro, Hidekazu Tanaka, Masahiro Tanabe, and Koji Shiraishi. Supervision: Hidekazu Tanaka, Masahiro Tanabe, and Koji Shiraishi.

## Funding

This clinical research received no specific grant from any funding agency in the public, commercial, or nonprofit sectors.

## Disclosure

All authors have read and agreed to the final version of the manuscript.

## Ethics Statement

The present work was carried out within the framework of the ethics committee/institutional review board.

## Conflicts of Interest

The authors declare no conflicts of interest.

## Data Availability

The data that support the findings of this study are available on request from the corresponding author. The data are not publicly available due to privacy or ethical restrictions.

## References

[1] https://www.nccn.org/professionals/physician_gls/pdf/prostate.pdf, National Comprehensive Cancer Network Guidelines Version 2.2025: Prostate Cancer.

[2] Dee E. C. , Nezolosky M. D. , Chipidza F. E. et al., Prostate Cancer-Specific Mortality Burden by Risk Group Among Men With Localized Disease: Implications for Research and Clinical Trial Priorities, Prostate. (2020) 80, no. 13, 1128–1133, 10.1002/pros.24041. 32659024

[3] Shimabukuro T. , Tanaka H. , Tanabe M. et al., Long-Term Clinical Outcomes of Radical Prostatectomy Versus Image-Guided and Intensity-Modulated Radiation Therapy for Prostate Cancer: A Retrospective and Comparative Study, Advances in Urology. (2025) 2025. 10.1155/aiu/6412793 PMC11952913 40161854

[4] Artibani W. , Porcaro A. B. , De Marco V. , Cerruto M. A. , and Siracusano S. , Management of Biochemical Recurrence After Primary Curative Treatment for Prostate Cancer: A Review, Urologia Internationalis. (2018) 100, no. 3, 251–262, 10.1159/000481438. 29161715

[5] Roach M. , Hanks G. , Thames H. et al., Defining Biochemical Failure Following Radiotherapy With or Without Hormonal Therapy in Men with Clinically Localized Prostate Cancer: Recommendations of the RTOG-ASTRO PHOENIX consensus Conference, International Journal of Radiation Oncology Biology Physics. (2006) 65, no. 4, 965–974, 10.1016/j.ijrobp.2006.04.029. 16798415

[6] Scher H. I. , Morris M. J. , Stadler W. M. et al., Trial Design and Objectives for Castration-Resistant Prostate Cancer: Updated Recommendation From the Prostate Cancer Clinical Trials Working Group 3, Journal of Clinical Oncology. (2016) 34, no. 12, 1402–1418, 10.1200/jco.2015.64.2702. 26903579 PMC4872347

[7] Shimabukuro T. , Ohmi C. , Baba C. , and Shiraishi K. , Overall Survival Evaluation of Prostate Cancer Patients Treated With Androgen Deprivation Therapy by Estimating Fluctuation Patterns of Metabolic Factor Serum Levels, Japanese Journal of Urology. (2022) 113, no. 1, 1–11, 10.5980/jpnjurol.113.1. 36682805

[8] Chen W.-J. , Kong D.-M. , and Li L. , Prognostic Value of ECOG Performance Status and Gleason Score in the Survival of Castration-Resistant Prostate Cancer: A Systematic Review, Asian Journal of Andrology. (2021) 23, no. 2, 163–169, 10.4103/aja.aja_53_20. 33159024 PMC7991808

[9] Halabi S. , Lin C.-Y. , Kelly W. K. et al., Update Prognostic Model for Predicting Overall Survival in First-Line Chemotherapy for Patients With Metastatic Castration-Resistant Prostate Cancer, Journal of Clinical Oncology. (2014) 32, no. 7, 671–677, 10.1200/jco.2013.52.3696. 24449231 PMC3927736

[10] Shore N. D. , Luz M. D. A. , Giorgi U. D. et al., Improved Survival With Enzalutamide in Biochemically Recurrent Prostate Cancer, New England Journal of Medicine. (2026) 394, no. 6, 563–575, 10.1056/nejmoa2510310. 41124201

